# Risk Factors and Spatial Distribution of *Schistosoma mansoni* Infection among Primary School Children in Mbita District, Western Kenya

**DOI:** 10.1371/journal.pntd.0002991

**Published:** 2014-07-24

**Authors:** Sachiyo Nagi, Evans A. Chadeka, Toshihiko Sunahara, Faith Mutungi, Yombo K. Dan Justin, Satoshi Kaneko, Yoshio Ichinose, Sohkichi Matsumoto, Sammy M. Njenga, Masahiro Hashizume, Masaaki Shimada, Shinjiro Hamano

**Affiliations:** 1 Department of Parasitology, Institute of Tropical Medicine, Nagasaki University (NUITM), Nagasaki, Japan; 2 Graduate School of Biomedical Science, Nagasaki University, Nagasaki, Japan; 3 Department of Vector Biology and Environment, Institute of Tropical Medicine, Nagasaki University (NUITM), Nagasaki, Japan; 4 Eastern and Southern Africa Centre of International Parasite Control (ESACIPAC), Kenya Medical Research Institute (KEMRI), Nairobi, Kenya; 5 Department of EcoEpidemiology, Institute of Tropical Medicine, Nagasaki University (NUITM), Nagasaki, Japan; 6 Nagasaki University Nairobi Research Station, NUITM-KEMRI Project, Nairobi, Kenya; 7 Department of Infectious Disease Control and International Medicine, Niigata University Graduate School of Medical and Dental Sciences, Niigata, Japan; 8 Department of Paediatric Infectious Diseases, Institute of Tropical Medicine, Nagasaki University (NUITM), Nagasaki, Japan; Centers for Disease Control and Prevention, United States of America

## Abstract

**Background:**

An increasing risk of *Schistosoma mansoni* infection has been observed around Lake Victoria, western Kenya since the 1970s. Understanding local transmission dynamics of schistosomiasis is crucial in curtailing increased risk of infection.

**Methodology/Principal Findings:**

We carried out a cross sectional study on a population of 310 children from eight primary schools. Overall, a total of 238 (76.8%) children were infected with *S. mansoni*, while seven (2.3%) had *S. haematobium*. The prevalence of hookworm, *Trichuris trichiura* and *Ascaris lumbricoides* were 6.1%, 5.2% and 2.3%, respectively. *Plasmodium falciparum* was the only malaria parasite detected (12.0%). High local population density within a 1 km radius around houses was identified as a major independent risk factor of *S. mansoni* infection. A spatial cluster of high infection risk was detected around the Mbita causeway following adjustment for population density and other potential risk factors.

**Conclusions/Significance:**

Population density was shown to be a major factor fuelling schistosome infection while individual socio-economic factors appeared not to affect the infection risk. The high-risk cluster around the Mbita causeway may be explained by the construction of an artificial pathway that may cause increased numbers of *S. mansoni* host snails through obstruction of the waterway. This construction may have, therefore, a significant negative impact on the health of the local population, especially school-aged children who frequently come in contact with lake water.

## Author Summary

It is estimated that more than ten percent of the world's population is at risk of schistosome transmission, with over 90% of infections occurring in sub-Saharan Africa. In Kenya, schistosomiasis remains a major public health concern particularly around Lake Victoria. The objective of this study was to identify the risk factors associated with *Schistosoma mansoni* infection among schoolchildren on the shores and adjacent islands of Lake Victoria in Mbita district, western Kenya. High local population density was identified as an important risk factor for *S. mansoni* infection. Socio-economic factors were not found to be significantly associated with infection risk. Our study suggests that environmental changes related to causeway construction and the dense human population around Mbita town may result in favourable ecological conditions for *S. mansoni* transmission.

## Introduction

Schistosomiasis is a parasitic disease affecting 249 million people worldwide. It is endemic in 78 countries with over 90% of cases occurring in sub-Saharan Africa [1]. About 779 million people, more than 10% of the world's population, were estimated to have been at risk of schistosome infection in mid-2003 [2]–[5]. In Africa, schistosomiasis is due predominantly to infection with *Schistosoma mansoni*, which causes intestinal schistosomiasis, and *Schistosoma haematobium* which causes urinary schistosomiasis [6].

Small scale spatial heterogeneity is a typical epidemiological feature of schistosomiasis [7], [8]. Such heterogeneity is closely associated with the distribution of the snail intermediate host, and with human contact with infective water [9], [10]. Past studies showed correlation between schistosome transmission and several epidemiological and socio-economic factors such as age [11], [12], sex, sources of drinking water, latrine availability, sanitation, hygiene [13]–[17], urbanization and population growth. [18]–[21]. Moreover, some works have merged both our understanding of demographic risk factors together with environmental transmission dynamics in order to create large scale (national, regional, continental) maps that are instrumental in designing control programmes for the disease [22]–[25], while small scale analysis is important in contributing to the local distinct need [26]–[28].

Schistosomiasis is increasingly a major health problem among communities around Lake Victoria. Geographical patterns of *S. mansoni* infection have been described in this area in relation to proximity to the lake [25], [29]–[31] and in comparison of islands versus mainland habitation, where risk is higher on the islands [32]. Identifying local risk factors of infection at multiple levels is crucial so as to understand how transmission varies within small spatial scales and how it changes over time. In addition, identifying risk factors may facilitate disease control by targeting high risk groups or by informing possible intervention strategies. The main objective of this study was to identify the risk factors associated with *S. mansoni* infection among schoolchildren in Mbita and the two adjacent islands (Rusinga and Ngodhe) of Lake Victoria, Kenya.

## Methods

### Ethics statement

The study was reviewed and approved by the scientific steering committee and ethical review committee of the Kenya Medical Research Institute, Kenya (KEMRI, SSC No. 2084), and the ethical review board of Institute of Tropical Medicine, Nagasaki University, Japan (No. 10121666). Written informed consent was obtained from parents/guardians and schoolchildren prior to the study. Children infected with schistosomes were treated with 40 mg/kg praziquantel and those infected with soil transmitted helminths (STHs) were treated with 400 mg albendazole by a clinical officer in accordance with WHO guidelines [33]. All children positive for malaria were treated with artemether/lumefantrine (AL) according to national guidelines for uncomplicated malaria [34]. A study feedback meeting was held with parents or guardians of participants, as well as the head masters and health teachers of the schools.

### Study area

**Figure 1:**
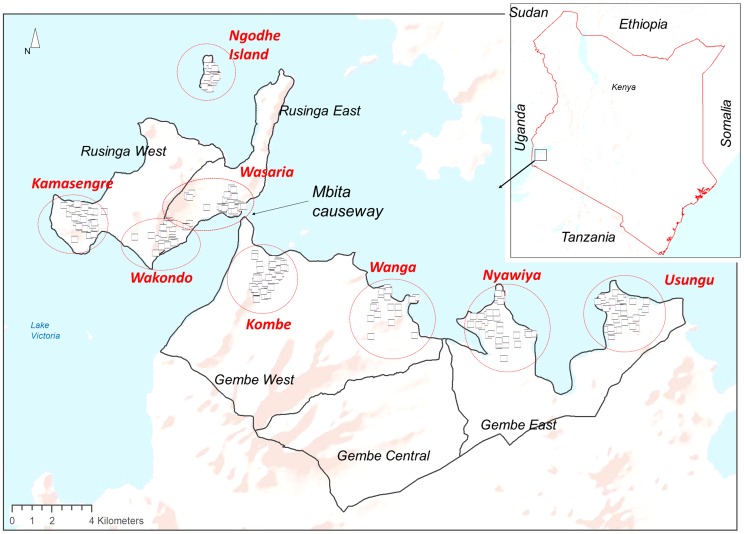
Map of the study area. A black rectangle in the national map indicates the position of the area shown in the main map frame. Small squares in the main frame indicate house of 310 schoolchildren. Dotted red circles indicate the extent of the area from which children attend the schools.

This study was conducted on the shores and islands of Lake Victoria in Nyanza province, Mbita district, western Kenya (Figure 1) in an area covered by a health and demographic surveillance system (HDSS) [35], [36]. The Mbita HDSS includes Rusinga east and west on the island and Gembe east and west on the mainland. Ongoing HDSS data showed that the total population in Mbita was 55,929 during our survey conducted in 2011. Notably, population density on Rusinga Island was twice as high as in the Gembe region. In Mbita district, the waterway separating Rusinga Island from the mainland was filled in 1985 and a road to Rusinga Island was constructed to facilitate transportation of people, goods and services. Economic activities are high around and within a 5 km radius from the centre of Mbita causeway, referred to as an urban area, while rest of the study area was treated as rural. Mbita is dominated mainly by the fishing communities living in the immediate vicinity of the lake. The temperature in Mbita ranges from 15°C to 30°C. Rain seasonality is bimodal with a short rainy season starting from October to December, while a longer rainy season lasts from March to May. The average annual rainfall ranges between 800–1,200 mm in the western part of the study area in Rusinga Island while Gembe receives slightly higher rainfall of 800–1,900 mm. In our study, HDSS data was used for obtaining household locations and population density.

### Study design

A cross-sectional study was conducted between September and November 2011. According to the education office in Mbita district, the primary school enrollment rate was 91.6%. The inclusion criteria of the schools were to be a full grade primary school and not to have received mass-chemotherapy for a year prior to the study. As most of the private primary schools received mass drug administration for STHs a year prior to the study, they were excluded. The schoolchildren in 4^th^ grade were targeted in this study and the total number of them were 1,747 in 2011. Of these, 888 were females and 859 were males. Among the 64 public primary schools, 39 schools met inclusion criteria and 8 schools were randomly selected as clusters (Figure 1). Parent/guardian and teacher association meetings were held in all selected schools prior to the survey for communicating the study purpose and obtaining their consent with full understanding. Ninety-eight percent of parents/guardians consented and consequently, 310 of all 4^th^ grade children were enrolled in the study.

### Sample collection

All children were instructed to provide stool specimens in a labeled specimen cup on three consecutive days. The school health teacher or class teacher guided students on stool sample collection during container distribution, a day before the survey. A trained field worker visited the school during morning break time with a registration sheet to ensure all students provided samples. Those who did not provide samples were followed up to ensure each child provided maximum possible samples. The Kato-Katz fecal thick smear technique was used for the detection and the quantification of *S. mansoni* eggs and the presence of STHs. Intensity of infection was estimated as the number of eggs per gramme of feces (epg) [37], . Slides were prepared and examined by two independent readers within an hour for hookworm egg detection and within 24 hours for the rest of the parasites in focus. Parasite eggs were counted and the arithmetic mean of 3 slides per child was calculated to give the intensity of infection. The extent of *S. mansoni* infection was categorized as light (1–99 epg), moderate (100–399 epg) or heavy (≥400 epg) according to WHO guidelines [33]. The intensity of infection per school was calculated as the geometric mean of egg excretion among all children testing within the school. In addition, the presence of *S. haematobium* and *Plasmodium* spp. were examined to assess their association with *S. mansoni* infection. Midday urine was collected for the detection of *S. haematobium* eggs using direct microscopy examination since *S. haematobium* is known not to be endemic in the study area [6]. Venous blood was collected for the microscopic examination of *Plasmodium* spp. by thick and thin Giemsa stained smears. Additional haematological and serological examinations were also carried out for a separate study.

### Questionnaire

To identify risk factors for *S. mansoni* infections, trained interviewers administered a questionnaire to children during the parasitological survey at the school, while parents/guardians were interviewed in the household setting. Information about individual treatment history for schistosomiasis and the water contact behaviour of each child was collected in the school setting. For socio-economic factors, the household head or the most informed adult present during the household interview gave information on: ownership of land, household size, total number of rooms in house, mother's/female guardian's education level and the main source of drinking water. The age of each child was confirmed by cross checking with official birth certificates or church baptism cards during household visits. In addition, an observation checklist was used to collect information on house structure, latrine and electricity availability in each household. Houses were categorized into two groups: traditional houses with grass roofs and modern houses with iron sheet or cemented roofs. The number of persons per room was obtained as one of the indicators of socio-economic status by dividing the household size by total number of rooms in the house. Households with more than two persons per room were categorized as overcrowded, since the average number of persons per room was two in the study population.

### Data analysis

Participants were defined as being positive for each helminthic infection if at least 1 egg was detected in their stool for *S. mansoni*, STHs or in urine for *S. haematobium*. For *P. falciparum* infection, both thick and thin blood smears were examined using a light microscope at ×100 with an oil immersion objective. Positive cases were defined as those with at least one malaria parasite detected in the microscopic field of 200 white blood cells for thick film or 2,000 red blood cells for thin film [39]. The intensity of helminth infection was expressed as the arithmetic mean of three slides per child, while the intensity of helminth infection per school was expressed as the geometric mean. Total egg counts of *S. mansoni* in fecal samples were analyzed in relation with potential risk factors by using both a generalized linear model (GLM) and a generalized linear mixed model (GLMM) with school as a random factor. Since fecal egg count is over-dispersed, a negative binomial generalized linear model (NB-GLM) and a negative binomial generalized linear mixed model (NB-GLMM) were used. The mixed model was employed to account for the potential lack of independence among samples that emerges from children attending the same school [40]. As children attending the same school were clearly clustered around the school, the school effects might be interpreted as the effects of the areas where children reside.

Local population density was obtained for each child using the HDSS population data. The number of people living within a radius of 1 km was counted for all participants' houses using Quantum GIS version 1.7.4. [41]. This scale was selected because population density showed the strongest association with *S. mansoni* infection when the radius was set at 1 km. The HDSS population data was incomplete for Ngodhe Island and therefore we used the total population of the island (449 persons; according to a local health staff), since most houses on the island were within 1 km from another participant's houses. The shortest straight-line distance from the study participants' house to the lake shore was obtained using Quantum GIS [41]. Spearman's rank correlation was used to test associations between prevalence and intensity of *S. mansoni* infection and distance to the lake. All statistical analyses were carried out using R version 3.0.1[42] and *P*-values less than 0.05 were considered significant. The glmmADMB package was applied for analysis of an over-dispersed continuous variable (infection intensity).

To examine whether the intensity of *S. mansoni* infection is spatially clustered, a spatial scan statistical treatment was applied to point data on household location using SaTScan software (version 9.1.1.) [43]. As models for over-dispersed count data are not available in SaTScan, we applied normal model to the log (N+1) transformed egg count. A purely spatial model was applied and a scan for areas with high values was performed. The maximum size of high-risk clusters was set to 50% of the total number of subjects. To evaluate statistical significance, 999 Monte-Carlo replications were conducted. To examine whether any spatial clusters could be explained by individual risk factors, a scan was also performed with the residual values of a linear regression model of log (N+1) with independent variables which were significantly associated by the egg count in the NB-GLM. The spatial clusters of the residuals can be interpreted as those adjusted for independent risk factors.

## Results

### Characteristics of the study participants

Demographic and socio-economic characteristics of the study participants are shown in Table 1. The study involved 310 fourth grade schoolchildren from eight schools, 138 (44.5%) of the children were male, and 172 female (55.5%). Their ages ranged from 9 to 19 years and the median age was 12 years for both sexes. The majority (81.0%) of the children lived in traditional houses with an average of 2–3 rooms. Most of the children (95.3%) lived in overcrowded houses, 95.8% had no electricity and 53.9% had no latrine. Over three quarters (76.4%) of families owned land and 84.8% of mothers/female guardians completed 4^th^ grade or further education. The majority of households (84.8%) used the lake as the main source of drinking water. Apart from one child, the rest of the children (99.7%) had routine lake water contact an average of 2–3 times per week mainly through bathing and domestic washing purposes. Individual treatment history of schistosomiasis was also confirmed by questionnaire and none of participants was treated at least one year before the study.

**Table 1:** Potential risk factors of *Schistosoma mansoni* infection.

Variable		Number (%)
Age	Range	9–19 years
	Median	12 years
Sex	Female	172 (55.5)
	Male	138 (44.5)
Population density	Median	950/km^2^
School	Ngodhe (Rusinga Island)	26 (8.4)
	Wasaria (Rusinga Island)	59 (19.0)
	Kamasengre (Rusinga Island)	36 (11.6)
	Wakondo (Rusinga Island)	28 (9.0)
	Kombe (main land)	47 (15.2)
	Wanga (main land)	26 (8.4)
	Nyawiya (main land)	47 (15.2)
	Usungu (main land)	41 (13.2)
Mother's education	No	47 (15.2)
(At least 4^th^ grade)	Yes	263 (84.8)
House structure	Traditional/Semi-permanent	251 (81.0)
	Permanent	59 (19.0)
Electricity	No	297 (95.8)
	Yes	13 (4.2)
Ownership of land	No	65 (24.4)
	Yes	211 (76.4)
Number of rooms	1–3 room (s)	261 (94.2)
	4–7 rooms	16 (5.8)
Crowding	No	13 (4.7)
(>2 rooms)	Yes	261 (95.3)
Water supply	Lake	263 (84.8)
	Rain	9 (2.9)
	Tap/Pipe	38 (12.3)
Latrine	No	167 (53.9)
	Yes	143 (46.1)
Lake water contact	No	1 (0.3)
	Yes	309 (99.7)

### High prevalence and intensity of *S. mansoni* among schoolchildren in Mbita

The overall prevalence of schistosomes, STHs and *P. falciparum* in each school is summarized in Table 2. More than three quarters (76.8%) of the students were infected with *S. mansoni*, while seven (2.3%) were infected with *S. haematobium*. All the children infected with *S. haematobium* were co-infected with *S. mansoni* and had previously stayed in areas endemic for *S. haematobium*, further inland from Lake Victoria. At least 12.6% of the schoolchildren were infected with one or more species of STHs. Prevalence of hookworm, *Trichuris trichiura* and *Ascaris lumbricoides* was 6.1%, 5.2% and 2.3%, respectively. Thirty-seven schoolchildren (12.0%) were infected with *P. falciparum*. A total number of 248 (80%) were infected with at least one of the examined parasites. Co-infection of *S. mansoni* with *P. falciparum* 13.1% (31/236) was the most common in the study area. In addition, multiple infections with more than three species were found in a few cases. There was no difference in males and females in co-infection of *S. mansoni* with *P. falciparum* (*P* = 0.52). All possible combinations for parasitic infections were found nearly in the expected numbers (data not shown), indicating neither synergistic nor antagonistic effects of polyparasitism.

**Table 2:** Prevalence of parasitic infections among schoolchildren, Mbita, Kenya.

School name	Overall prevalence		Rusinga Island			Ngodhe Island	Gembe (mainland)			
	cases/total (%)	95%CI* (%)	Wasaria n = 59 (%)	Wakondo n = 28 (%)	Kamasengre n = 36 (%)	Ngodhe n = 26 (%)	Kombe n = 47 (%)	Wanga n = 26 (%)	Nyawiya n = 47 (%)	Usungu n = 41 (%)
*Schistosomes*										
*S. mansoni*	238/310 (76.8)	72.1–81.5	58 (98.3)	26 (92.9)	31 (86.1)	15 (57.7)	39 (83.0)	21 (80.8)	35 (74.5)	13 (31.7)
*S. haematobium*	7/309 (2.3)	0.6–3.9	0 (0)	1 (3.6)	1 (2.8)	0 (0)	4 (8.5)	0 (0)	0 (0)	1 (2.4)
Soil transmitted helminths (STHs)	39/310 (12.6)	8.9–16.3								
*Hookworm*	19/310 (6.1)	3.5–8.8	2 (3.4)	1 (3.6)	0 (0)	5 (19.2)	2 (4.3)	3 (11.5)	5 (10.6)	1 (2.4)
*T. trichiura*	16/310 (5.2)	2.7–7.6	5 (8.5)	2 (7.1)	0 (0)	2 (7.7)	4 (8.5)	0 (0)	3 (6.4)	0 (0)
*A. lumbricoides*	7/310 (2.3)	0.6–3.9	3 (5.1)	1 (3.6)	1 (2.8)	1 (3.8)	0 (0)	1 (3.8)	0 (0)	0 (0)
*P. falciparum*	37/308 (12.0)	8.4–15.6	9 (15.3)	2 (7.1)	4 (11.1)	2 (7.7)	5 (10.6)	3 (11.5)	9 (19.1)	3 (7.3)

95% CI: 95% confidence interval.

The prevalence of *S. mansoni* differed significantly between schools, ranging from 31.7 to 98.3 percent, (Pearson chi-square test *P*<0.001). Age was not associated with prevalence of *S. mansoni* in this study. There was no significant difference between males and females for prevalence of any examined parasitic infections. Table 3 shows the intensity of *S. mansoni* infection in each school. Among those who were positive for *S. mansoni* eggs, the geometric mean number of eggs excreted per gramme of feces (epg) varied from 2.0 to 303.5 epg between schools. The overall mean intensity of *S. mansoni* infection was 207 epg with inter quartile range of 8 to 214 epg. The intensity of *S. mansoni* infection was categorized according to the WHO guidelines [33], children with light, moderate and heavy infections were 110 (35.5%), 78 (25.2%) and 50 (16.1%) respectively.

**Table 3:** Intensity of *Schistosoma mansoni* infection among schoolchildren in Mbita, Kenya.

School name	Rusinga Island			Ngodhe Island	Gembe (mainland)				Total *n* = 310 (%)
	Wasaria *n* = 59 (%)	Wakondo *n* = 28 (%)	Kamasengre *n* = 36 (%)	Ngodhe *n* = 26 (%)	Kombe *n* = 47 (%)	Wanga *n* = 26 (%)	Nyawiya *n* = 47 (%)	Usungu *n* = 41 (%)	
Intensity of *S. mansoni* (epg *)	303.5	116.4	72.8	9.7	34.9	27.2	15.6	2.0	n/a
Negative	1 (1.7)	2 (7.1)	5 (13.9)	11 (42.3)	8 (17.0)	5 (19.2)	12 (25.5)	28 (68.3)	72 (23.2)
Light (1–99 epg)	5 (8.5)	8 (28.6)	11 (30.6)	9 (34.6)	25 (53.2)	15 (57.7)	26 (55.3)	11 (26.8)	110 (35.5)
Moderate (100–399 epg)	25 (42.4)	11 (39.3)	10 (27.8)	6 (23.1)	13 (27.7)	4 (15.4)	7 (14.9)	2 (4.9)	78 (25.2)
Heavy (>400 epg)	28 (47.5)	7 (25.0)	10 (27.8)	0	1 (2.1)	2 (7.7)	2 (4.3)	0	50 (16.1)

epg: egg per gramme of feces; Intensity of *S. mansoni* infection was calculated using geometric mean.

**Figure 2:**
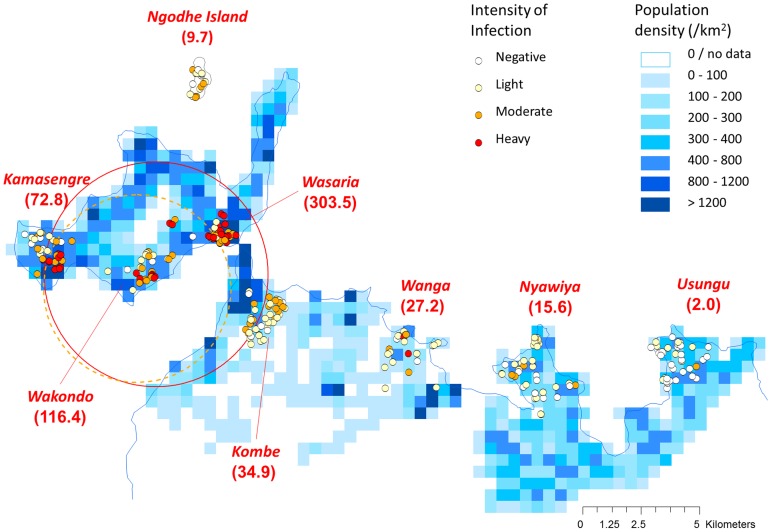
Intensity of *S. mansoni* infection among primary school children in Mbita, Kenya. The intensity of *S. mansoni* infection was mapped at the household level. According to the WHO guidelines, *S. mansoni* infection was categorized as: negative, light (1–99 epg), moderate (100–399 epg) or heavy (≥400 epg) infection. The number in the parentheses shows the geometric mean of the number of eggs in each school. Population density of each 500 m grid is shown in the background. The red solid circle indicates the unadjusted high risk cluster whereas the orange dotted circle indicates the cluster adjusted for potential risk factors as follows: sex, population density, house structure and latrine availability.

At the school level, the intensity of *S. mansoni* infections was strongly correlated with its prevalence (Spearman's rank correlation, rho = 0.98, *P*<0.001). The four schools with high prevalence and intensity of *S. mansoni* infection (Wasaria, Wakondo, Kamasengre, and Kombe) were aggregated around the bay in the west side of Mbita causeway (Figure 2). The mean of log (N+1) transformed egg counts were significantly different between inside and outside of clusters as 5.23 and 2.55, respectively (common estimate for standard deviation, 1.94; *P* = 0.001).

### Risk factors of *S. mansoni* infection

Table 4 shows the results of a bivariate analysis on the association between the intensity of *S. mansoni* infection and the potential risk factors with no consideration of school effects (NB-GLM). This indicated that males were more intensely infected than females (marginally significant). Several household-based factors also showed a significant association with high infection risk; houses in areas with higher population density, permanent houses and houses with latrine.

**Table 4:** Bivariate negative binomial generalized linear model (NB-GLM) for *Schistosoma mansoni* infection risk among schoolchildren Mbita, Kenya.

Parameter		Estimate	Std. Error	Z value	Pr (>|Z|)
Age		0.12964	0.06907	1.877	0.061
Sex	Male	0.4495	0.2328	1.931	0.054
Population density		4.171e-04	6.534e-05	6.383	1.73e-10***
Mother's education	Yes	−0.6236	0.4960	−1.257	0.21
House structure	Permanent	0.9139	0.2916	3.134	0.0017**
Light	Yes	0.3629	0.5799	0.626	0.53
Land	Yes	−0.1797	0.2909	−0.618	0.54
Number of rooms	>3 rooms	0.6911	0.5260	1.314	0.19
Crowding	Yes	−0.3731	0.5803	0.580	0.52
Latrine	Yes	0.6231	0.2310	0.231	0.0070**
Water supply*	Rain	−0.88838	0.69312	−1.282	0.20
(*Reference = Lake)	Tap/Pipe	0.02362	0.35475	0.067	0.95
Distance to the lake		−0.0004780	0.0002663	−1.795	0.073

Spatial scan statistics was performed for the residuals of the regression model of log (N+1) transformed egg count. The variables that were found to be significant and/or marginally significant in the NB-GLM (sex, population density, house structure and houses with latrine; orange dotted circle in Figure 2) were included in the calculation. A significant high-risk cluster occurred in a similar location to the unadjusted cluster (red circle in Figure 2) although the size of the cluster was smaller (radius of 4,006 meters; 53 children were included). The adjusted cluster included all the children of Wakondo and some of the children of Wasaria and Kamasengre but did not include any of the children living in Kombe. The mean of the residuals was significantly higher inside (1.50) than outside the cluster (−0.31), common estimate of standard deviation, 1.92; *P* = 0.001.

**Figure 3:**
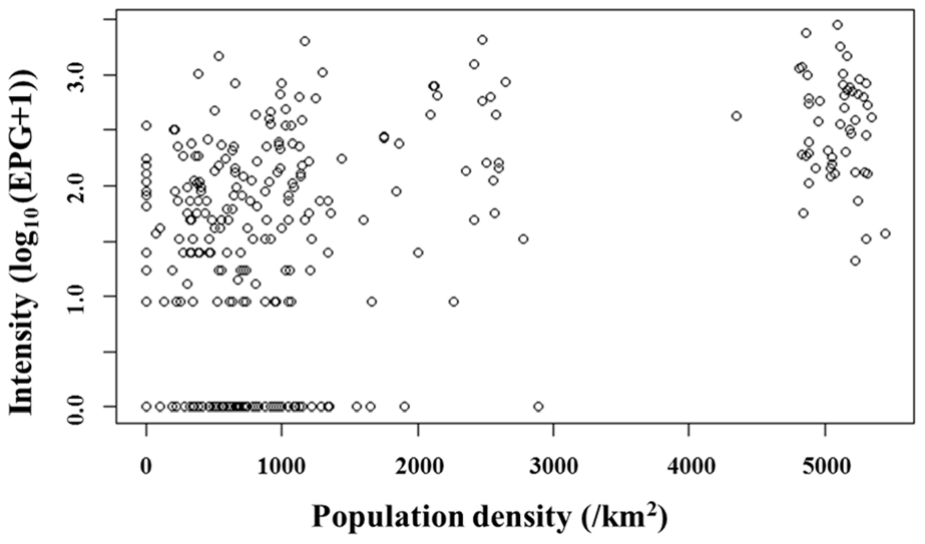
Relationship between local population density and intensity of *S. mansoni* infection. Scatter plot showing positive association between intensity of *S. mansoni* infection and population density. The population density and the intensity were expressed as the number of people within a radius of 1 km and log_10_ (epg+1), respectively.

When the school areas were included as random factor in a NB-GLMM, the effects of sex, house structure and latrine became non-significant (Table 5). Local population density was the only statistically significant factor for *S. mansoni* infection in the NB-GLMM (Table 5; *P* = 0.011). The association between population density and intensity of *S. mansoni* infection is further depicted in the map and scatter plot as shown in Figure 2 and 3. Evidently, population density is an independent factor influencing risk and intensity of *S. mansoni* infection, while evaluated socio-economic factors appeared not to affect the risk and intensity of *S. mansoni* infection in this study area.

**Table 5:** Bivariate negative binomial generalized linear mixed model (NB-GLMM) for *Schistosoma mansoni* infection risk among schoolchildren Mbita, Kenya.

Parameter		Estimate	Std. Error	Z value	Pr (>|Z|)
Age		0.0711	0.0667	1.06	0.29
Sex	Male	0.232	0.221	1.05	0.29
Population density		0.000302	0.000118	2.55	0.011*
Mother's education	Yes	−0.755	0.472	−1.60	0.11
House structure	Permanent	0.262	0.304	0.86	0.39
Light	Yes	0.479	0.535	0.9	0.37
Land	Yes	−0.180	0.291	−0.62	0.54
Number of rooms	>3 rooms	0.365	0.488	0.75	0.45
Crowding	Yes	−0.166	0.535	−0.31	0.76
Latrine	Yes	0.230	0.226	1.02	0.31
Water supply*	Rain	−1.059	0.632	−1.68	0.094
(*Reference = Lake)	Tap/Pipe	−0.100	0.387	−0.26	0.80
Distance to the lake		−0.000415	0.000324	−1.28	0.20

## Discussion

This study goes some way towards elucidating the risk factors associated with *S. mansoni* infection among schoolchildren in Mbita district, western Kenya. The prevalence of *S. mansoni* was high in almost all the schools sampled and more than three quarters (76.8%) of children were infected with *S. mansoni*. This result is consistent with the previous reports [32], [44], in which they showed an increased risk of *S. mansoni* infection compared to the early 1970s [45], [46], at which time the prevalence of *S. mansoni* was less than 50% among schoolchildren along the shores and islands of Lake Victoria in Mbita.

In a bivariate NB-GLM analysis, children living in permanent houses with latrine were infected with larger numbers of *S. mansoni* eggs. However, the statistical significance of these effects did not remain in the model when considering school effects as a random factor (NB-GLMM). This was considerable based on previous studies which showed higher infection risk associated with lower socioeconomic status [47]. We attribute this to the fact that families living in permanent houses with latrine tended to be found in densely populated areas where infection risk was high, since the local habit of defecation along lake shore is common even though the most of families living around town centre have latrines [28]. In the result, the residential location was closely associated with *S. mansoni* infection risk. This finding corroborates previous research by Booth and colleagues, which clearly indicated that environmental living circumstances were tightly connected with infection status and disease burden. In short, environmental exposure due to residential location rather than some fixed characteristics of an individual determines risk of infection [48]. Several studies have reported high risks of *S. mansoni* infection among people living close to a permanent water body [25], [29]–[31]. However, the effect of the distance to the lake was not significant in the present study. This could be the result of a small range of proximity to the lake, as the schools surveyed were all located within 1.0 km, and the children lived within 2 km, of Lake Victoria.

Population density was the single most important factor associated with *S. mansoni* infection risk on the shores and islands of Lake Victoria. Theoretically, the basic reproductive number (R_0_) of schistosomiasis linearly increases with human density. This is due to the fact that the rate of infection among snails depends on the absolute number, not the prevalence of infected hosts [49]. Thus higher infection risk in densely populated areas can be explained purely by numerical dynamics of transmission. In addition, higher nutritional load via domestic waste water from densely populated areas might enhance population growth of the snails [50]. We can therefore strongly suggest that the increase in population density in recent decades may partially explain the increase in *S. mansoni* prevalence in this area.

Notably, our study revealed the highest prevalence and intensity of *S. mansoni* infection was around the Mbita causeway. There was a tendency that infection risk decreased towards the eastern part of the mainland (Figure 2). Additionally, infection risk was very high in the three schools on Rusinga Island but not on Ngodhe Island. Our results suggest that a simple dichotomy like island-mainland comparison may obscure micro-geographical heterogeneity in *S. mansoni* transmission. This calls for additional ecological and environmental survey to understand the distribution and population dynamics of snail intermediate host which directly relates with the transmission of schistosomes. Spatial analysis indicated a high-risk cluster that includes the town center, the causeway and nearby villages in Rusinga. The high risk of infection in these areas could be partially explained by local population density. However, a significant high-risk cluster remained in a similar location even after adjustment for the effects of local population and other potential risk factors. Therefore, an aggregated risk factor that was not measured in the present study may exist in the west side of Mbita causeway. The construction of Mbita causeway in the 1980s has likely impacted the ecosystem surrounding Rusinga and the mainland, by promoting population activities, restricting water circulation and free movement of aquatic biota through blockage of the natural channels [51]. Such a change in ecological conditions may be one of the reasons why *S. mansoni* prevalence has drastically increased compared with 1970s, before the causeway construction [52],[53].

To conclude, increased risk of *S. mansoni* infection was observed in Mbita along the shores and islands of Lake Victoria. Moreover, the infection risk of *S. mansoni* was associated with high population density and was concentrated around the Mbita causeway. Urgent intervention efforts should be considered in order to reduce morbidity and mortality due to *S. mansoni* infection, taking into consideration region-specific risk factors for disease transmission.

## Supporting Information

Checklist S1 **STROBE checklist.**
(PDF)
