# Correction: Risk Factors and Spatial Distribution of *Schistosoma mansoni* Infection among Primary School Children in Mbita District, Western Kenya

**DOI:** 10.1371/journal.pntd.0003190

**Published:** 2014-08-28

**Authors:** The PLOS Neglected Tropical Diseases Staff

There is an error in the Acknowledgements section. Kentaro Kato did not contribute to this study. The correct Acknowledgements should read:

The authors appreciate teachers, parents and schoolchildren who participated in this study. We are grateful for the support offered by Mbita District hospital and District education office. Special thanks go to Janet Mbinya and Julius Andove for their laboratory investigations. We thank all staffs and field workers of Mbita Health and Demographic Surveillance System (HDSS) who guided us during our household visits. Gratitude is expressed to Noboru Minakawa, Gabriel O Dida, Carolyn L. Keogh and Richard Culleton for their thoughtful comments and inputs in the development of this manuscript. We deeply appreciate Ayub V. Ofulla for his kind support and arrangement to have former related study reports in study area. We also thank for administrative support from Tomoko Takaya, Hiromi Oda and the staff members of the NUTIM-KEMRI project. This paper is published with the permission of the Directory of KEMRI.

## References

[1] Nagi S, Chadeka EA, Sunahara T, Mutungi F, Justin YKD, et al (2014) Risk Factors and Spatial Distribution of *Schistosoma mansoni* Infection among Primary School Children in Mbita District, Western Kenya. PLoS Negl Trop Dis 8(7): e2991 doi:10.1371/journal.pntd.0002991 25058653 10.1371/journal.pntd.0002991 PMC4109881

